# Connective tissue growth factor is not necessary for haze formation in excimer laser wounded mouse corneas

**DOI:** 10.1371/journal.pone.0172304

**Published:** 2017-02-16

**Authors:** Xiaodi Feng, Liya Pi, Sriniwas Sriram, Gregory S. Schultz, Daniel J. Gibson

**Affiliations:** 1 Institute of Wound Research, University of Florida, Gainesville, Florida, United States of America; 2 Department of Pediatrics, University of Florida, Gainesville, Florida, United States of America; 3 Department of Biomedical Engineering, University of Florida, Gainesville, Florida, United States of America; Cedars-Sinai Medical Center, UNITED STATES

## Abstract

We sought to determine if connective tissue growth factor (CTGF) is necessary for the formation of corneal haze after corneal injury. Mice with post-natal, tamoxifen-induced, knockout of CTGF were subjected to excimer laser phototherapeutic keratectomy (PTK) and the corneas were allowed to heal. The extent of scaring was observed in non-induced mice, heterozygotes, and full homozygous knockout mice and quantified by macrophotography. The eyes from these mice were collected after euthanization for re-genotyping to control for possible Cre-mosaicism. Primary corneal fibroblasts from CTGF knockout corneas were established in a gel plug assay. The plug was removed, simulating an injury, and the rate of hole closure and the capacity for these cells to form light reflecting cells in response to CTGF and platelet-derived growth factor B (PDGF-B) were tested and compared to wild-type cells. We found that independent of genotype, each group of mice was still capable of forming light reflecting haze in the cornea after laser ablation (p = 0.40). Results from the gel plug closure rate in primary cell cultures of knockout cells were not statistically different from serum starved wild-type cells, independent of treatment. Compared to the serum starved wild-type cells, stimulation with PDGF-BB significantly increased the KO cell culture’s light reflection (p = 0.03). Most interestingly, both reflective cultures were positive for α-SMA, but the cellular morphology and levels of α-SMA were distinct and not in proportion to the light reflection seen. This new work demonstrates that corneas without CTGF can still form sub-epithelial haze, and that the light reflecting phenotype can be reproduced in culture. These data support the possibilities of growth factor redundancy and that multiple pro-haze pathways exist.

## Introduction

Based on observations from initial testing in cell culture systems, connective tissue growth factor (CTGF) was demonstrated to possess pro-fibrotic activities, and possibly to be a necessary factor for transforming growth factor-β’s (TGF-β) well-known pro-fibrotic activities[1–13]. By one hypothesis, the activity required the actions of yet other growth factors, resulting in a hypothetical combinatorial cell signaling mechanism[14]. Others have demonstrated in similar models that platelet-derived growth factor (PDGF) possess the said-same activities[15–20], but most research has focused on the chemotactic activities as opposed to cellular differentiation. What remains to be demonstrated is whether these activities are redundant, a part of a parallel signaling system, or if the previously observed effects are unique to cell culture systems. In order to begin determining the role of these growth factors in haze formation *in vivo*, gene-targeted approaches have been implemented to try and ascertain the necessity of one or another of these factors, or even several of them simultaneously[21, 22].

Previously, we reported the timing and localization of CTGF expression in wounded mouse and rabbit corneas with the intent of better understanding the potential role of CTGF, potential side-effects of its absence, and to improve gene-targeted approaches by knowing when, where, and for how long these therapies need to be active[8]. We have primarily focused on developing and testing RNAi-based approaches to test CTGF’s role in fibrosis[1, 21–24]. While we have had some promising results, the combination of testing both vector and therapy has been too cumbersome. We sought a model to ultimately test the necessity of CTGF for corneal scarring prior to continuing the development of the viral and non-viral vectors delivering RNAi therapies to improve corneal healing. In order to accomplish this, we began testing with a mouse model which possesses a floxed CTGF exon and a tamoxifen-inducible Cre recombinase[8]. The initial results from this model revealed the epithelial healing was delayed, but not prevented. The effect was statistically significant, but the magnitude of the delay was slight; suggesting that CTGF isn’t necessary for re-epithelialization, but that it does aid in more efficient closure.

In the time since we first formed our initial CTGF stimulated fibrosis hypothesis, others have demonstrated a variety of non-fibrotic functions for CTGF[25]. Our own discoveries have revealed CTGF’s expression in many tissues of the normal, unwounded, eye[8]; suggesting a more generic role for CTGF in normal tissue homeostasis. These data, taken all together, indicate a less fibrosis-specific activity for CTGF; meaning that while the process of fibrosis might make use of CTGF’s activity, other normal processes appear to do so as well. The immediate implication is that the risk of side-effects is now more certain, and in the case of re-epithelialization, demonstrated[8]. With this in mind, the presence of side-effects does not preclude the therapeutic potential for a treatment, it does however, add additional constraints and does necessitate a balanced discussion surrounding the cost versus benefit of a proposed treatment.

In the work reported herein, we sought to determine if CTGF was necessary for haze formation, and employed a model with the most complete ablation of the protein. Such an approach traded the size and biological human-like similarities of rabbit corneas for the power of control over our protein of interest (CTGF). At the outset of this project, it was well known that mouse corneas do not form haze as frequently as rabbits after a partial thickness excimer laser wound. While the limits of the animal model were known, we decided to both test the *in-vivo* injury model as well as to establish primary cell cultures of the model mouse corneal fibroblasts. In the event that the *in-vivo* work was inconclusive, we could still test the necessity of endogenously produced CTGF for the fibroblasts to respond to other known pro-fibrotic factors using the *in-vitro* models used to test these growth factors’ activities to date.

## Materials and methods

### Mice

All animals used in experiments reported herein were treated in a manner consistent with the ARRIVE guidelines and were carried out in accordance with the National Institutes of Health guide for the care and use of Laboratory animals (NIH Publications No. 8023, revised 1978). The protocols used to obtain the data presented herein were reviewed and approved by the University of Florida Institutional Animal Care and Use Committee. Briefly, the mice were observed daily after surgery by the study staff in addition to the daily rounds of the UF Animal Care Services. Mice judged to be moribund by a veterinarian would be immediately euthanized via cervical dislocation under general anesthesia with inhaled isoflurane, followed by confirmation of death by thoracotomy. In the course of these experiments, no mice became ill, were moribund, or died unexpectedly.

The conditional CTGF knockout mouse strain that was previously reported[8] were used in these experiments. Genotyping for *Ctgf* was carried out using primers 5’ CTGTTCTAAGACCTGTGGGATG 3’ (sense) and 5’ GCCCTTCTAAGGAAGACAGAAG 3’ (antisense). Wild-type mice, floxed but non-induced, heterozygous knockout mice, and homozygous mice underwent excimer laser surgery on their corneas as described before[8]. Prior to euthanization, the eyes were exposed and a macrophotograph was taken of the eye to record the haze[26]. The images were globally adjusted to make the retina black and to improve contrast using ImageJ’s “Brightness & Contrast” adjustment using the same manual setting propagated to all images (uniform global adjustment).

#### *Post-hoc* genotyping of the eye

To monitor the efficacy of knockout and to observe the degree of Cre mosaicism, the eyes were enucleated, fixed, and embedded in OCT media. Between 300 to 400 μm of transverse tissue sections up until the middle of the eye were collected and the genomic DNA (gDNA) was extracted using QIAamp DNA FFPE Tissue Kit (Qiagen, Inc., Valencia, CA, USA). The gDNA was then used to genotype the eye of the mice ensuring an accurate account of the tissue genotype to pair with the haze outcome using primers 5’ AATACCAATGCACTTGCCTGGATGG 3’ (sense) and 5’ GAAACAGCAATTACTACAACGGGAGTGG 3’ (antisense).

### Primary corneal fibroblast cell culture

Briefly, cell cultures of mouse corneal fibroblasts and CTGF knockout mouse corneal fibroblasts were established in a gel plug containing cell culture plate(Cell Biolabs, Inc., San Diego, CA, USA). The goal was to replicate the *in vivo* injury conditions including allowing the cells to become confluent and then “injuring” them with a circular defect upon removal of the gel plug. The cells were grown in high glucose DMEM (Corning Cellgro DMEM, Mediatech, Inc., Manassas, VA, USA) supplemented with 10% FBS and antibiotic/antimycotic (Gibco, Thermo Fisher Scientific, Waltham, MA, USA) until nearly confluent around the gel plug. The gel plug was removed per the manufacturer’s instructions and the cells were treated either with serum starvation, 25 ng/ml PDGF-BB (PeproTech: Rocky Hill, NJ, USA), or 250 ng/ml CTGF (US Biological, Salem, MA, USA).

#### Wound healing model

The wells were imaged on a standard transmission mode inverted microsope at 6 different time points over a 65 hour period. The images were opened in ImageJ and the remaining hole was quantified and the % area remaining open was calculated (i.e. time 0 = 100% open).

#### Light reflection phenotype

The cells were incubated for 3 days prior to imaging. The cells were washed in PBS and stored in PBS until imaging. The plate was placed on top of a low reflection black cloth. A macrophotograph was taken in the same manner done *in-vivo*. The PBS was removed from the cells and replaced with methanol to dehydrate and thereby mitigate any potential turbidity in the cells. The cells were then re-imaged.

#### α-smooth muscle actin staining

After the initial photographic imaging of the wells, the wells were fixed with 10% neutral buffered formalin for 1 hour. The wells were again photographed as before. Each well was blocked with 10% normal goat serum (Vector Labs, Burlingame, CA). The wells were stained by direct immunofluorescence with a Cy3 labeled mouse monoclonal antibody to α-SMA (Clone 1A4, Sigma-Alderich, St. Louis, MO). The wells were washed 3 times prior to counterstaining with DAPI. The wells were permanently mounted with hardening glycerol jelly and then imaged on an inverted cell-culture microscope via epifluorescent imaging.

#### Haze quantification

The images taken of the mouse corneas and the cell culture wells were quantified for light reflection using a previously reported method [26]. For the cell culture, the serum starved wild-type wells were averaged and used as background subtraction for the other wells. The numbers represent how much more light was reflected by the experimental wells compared to the serum starved wild-type cells.

### Statistical analysis

For all quantitative analyses, a one-way analysis of variance (ANOVA) was performed using Microsoft Excel’s Data Analysis Pack using an α = 0.05. Based upon the resulting observation of highly unequal variances, Tukey’s Honest Signficant Difference test was not considered appropriate. Instead, a *post hoc* analysis using Fisher’s Least Significant Difference (LSD) was performed via multiple unpaired, two-tailed Student’s t-Tests with unequal variance. A threshold for statistical significance was set to p < 0.05. Fisher’s LSD test carries with it a higher risk of false positives. To mitigate this risk, we have used the imaging and micrography data to validate potentially significant findings.

## Results

### In-vivo haze formation

**Fig 1:**
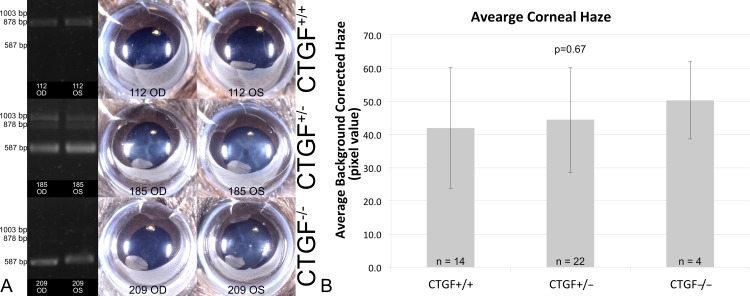
CTGF KO mouse corneas still form haze. A) *Post hoc* genotyping results in the injury corneas reveals good recombination in the homozygous KO corneas, but some degree of Cre-mosaicism in the heterozygous corneas. B) Quantitative analysis via macrophotography reveals increase haze in the absence of CTGF. An ANOVA did not detect any statistical significance among the groups.

The wild type and homozygous knockout had clear genotypes whereas the heterozygous KO had some degree of mosaicism (Fig 1), with some residual floxed allele remaining intact. The key finding, however, is that even in the absence of full-length CTGF, the mouse corneas can still form light reflecting haze (Fig 1). The images shown are the worst from each group, but a quantitative analysis revealed an increased average haze in heterzygotes (+2.3 pixel units vs. wild-type) and even higher in full knockouts (+8.3 pixel units vs. wild-type), though the ANOVA did not find any statistically significant differences among the groups.

### Growth factor response *in vitro*

**Fig 2:**
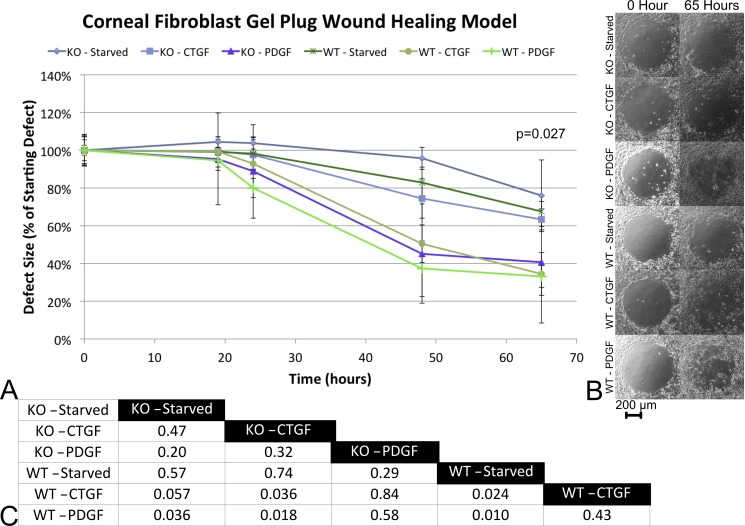
CTGF KO *versus* wild-type primary corneal fibroblast cultures. A) The rate at which primary fibroblasts can cover a circular defect in a monolayer culture varies by cell type. B) Representative images for each of the test conditions at the first (left) and last (right) time point. C) The results of *post hoc* comparisons among the test conditions.

A primary cell culture model in a gel-plug well was used to model both wound closure and light reflection. An ANOVA found significant differences among the groups at the 65 h time point. *Post hoc* analysis of the time course found no difference between either cell type when serum starved. The wild-type cells significantly increased wound closure rates in response to either CTGF (p = 0.02) or PDGF-BB (p = 0.01, Fig 2). The application of either CTGF of PDGF-BB did not statistically improve wound closure on the CTGF KO cells.

**Fig 3:**
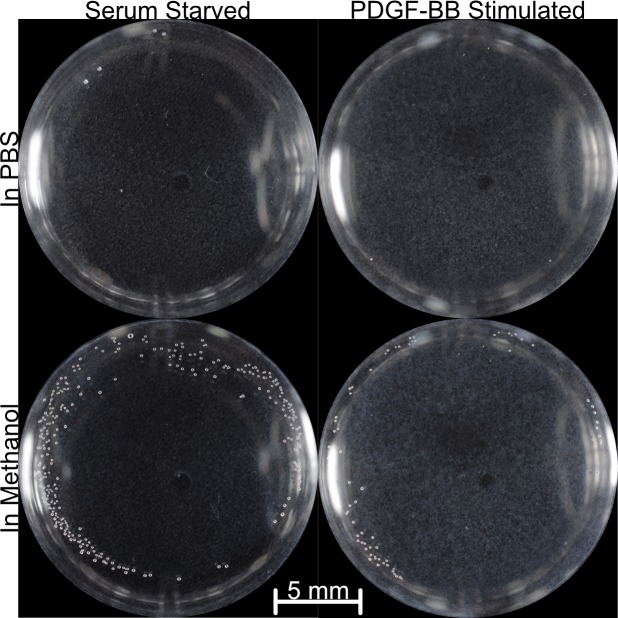
Pathological light reflection can be modeled *in vitro* in response to growth factor stimulation. The light reflection is not grossly affected by immersion media, though those submersed in methanol did have more bubbles (small white dots at the periphery of the well).

Since the mouse corneas still formed haze, we sought to determine if the fibroblasts had any differential sensitivity to known pro-fibrotic factors. We sought, for the first time, to determine if the cell cultures possess the pathological light reflection seen *in-vivo*. While α-SMA is used as a molecular marker for myofibroblasts in general, and sub-epithelial haze in the cornea, it is ultimately light reflection of the cells that is pathological[27]. We found that the cells could reflect light in a manner reminiscent of sub-epithelial haze, and that either PBS or methanol were amenable to imaging (Fig 3).

**Fig 4:**
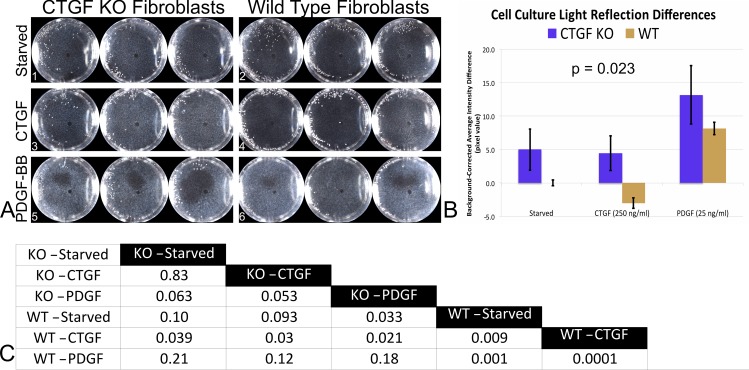
Changes in light reflection between the two cell types when stimulated by growth factors. A) A globally enhanced image of the replicated treatment wells showing grossly more light reflection in the PDGF-BB treated wells. B) Light scatter quantities normalized to wild type serum starved cells, with the results of the ANOVA. C) The p-value results from the *post hoc* comparisons among the treatment wells and cell types.

The CTGF KO primary corneal fibroblasts and wild type corneal fibroblasts were similarly treated with serum starvation, CTGF, or PDGF-BB (n = 3 each), and the wells were then inspected 3 days after treatment (Fig 4A). The results from a one-way ANOVA found that there was a significant differences among these groups (p = 0.023, Fig 4B). In comparing the WT cells and KO cells, the average amount of light reflection was higher in the CTGF KO cells (Fig 4B). However *post hoc* analysis (Fig 4C) revealed that the PDGF-BB treated cells remained a statistical trend (p = 0.18). In comparison to serum starved wild type cells, most wells were statistically different, with starved wild type cells trending towards being more reflective (p = 0.10) and CTGF treated cells trending towards being less reflective (p = 0.09).

**Fig 5:**
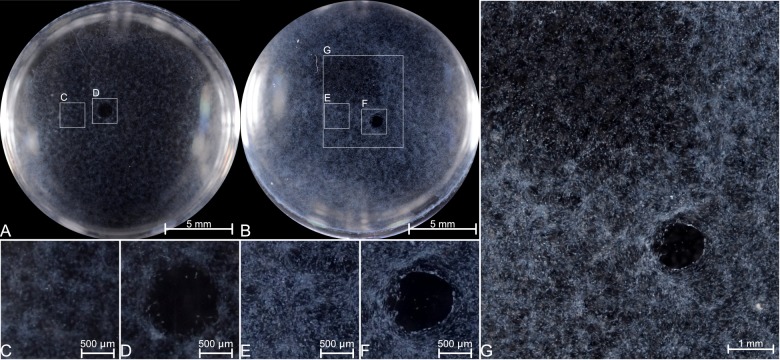
Light reflecting cells qualitative differences. These images were equivalently globally enhanced for better visualization. A) serum starved cells and b) PDGF-BB treated. C&E) the light reflective cells in the main body of the wells. D&F) the remaining gel-plug hole which has yet to be completely closed. G) A detailed image of the 3 distinct portions of the PDGF-BB treated wells which shows very haze-like cell phenotype which surrounds cells without such a phenotype (upper-left), in contrast to the still acellular hole.

While both CTGF and PDGF-BB treated wells were grossly more reflective than the control (Fig 4), the PDGF-BB treated cells are more densely opaque. When viewed in higher detail (Fig 5), the PDGF-BB treated cells have structural features that are more consistent with sub-epithelial haze. Most striking is the appearance of a spreading-like phenotype, with regions in each well appearing to not yet be affected. This pattern may represent a propagation of phenotype in a time-dependent manner.

The role of CTGF in fibrosis has largely been supported by its *in vitro* activities. Other growth factors, such as PDGF-BB, have been demonstrated to possess the same activity *in vitro*. Wild-type cells and CTGF KO cells still possess the capacity to respond to full length CTGF as well as PDGF-BB (Fig 3). The response to PDGF-BB was greater given that an approximately 6.6-fold lower molar amount produced a visually more discernable amount of light reflection.

Detailed examination of the light reflection in the untreated well (Fig 5A) versus the PDGF-BB treated well (Fig 5B) shows that they are very different. The non-treated cells appear, at most, to be like slightly opaque clouds (Fig 5C) while the PDGF-BB treated cells are highly reflective (Fig 5E). We are not yet certain if the few cloudy cells are spontaneously formed myofibroblasts, and their cloudiness is due to poor focus, or if they represent another phenotype. Both wells share the common feature of the residual gel plug area that has yet to be occupied by cells (Fig 5D and 5F). There is a region in the PDGF-BB treated well that had not yet taken on the highly filamentous and reflective phenotype (top-left of Fig 5G) and grossly looks similar to the untreated well. This patch of low reflectivity gives the appearance that the reflective phenotype also propagates by spreading[26], but that the process was incomplete at this time point.

**Fig 6:**
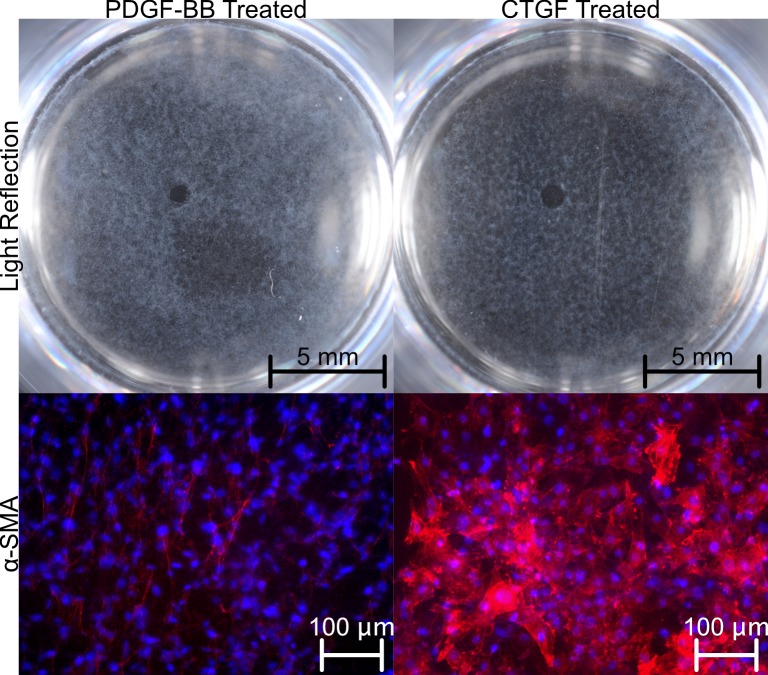
Light reflection and α-SMA levels are not correlated. The phenotypic nature is grossly similar in that both reflect light and have α-SMA, but the arrangement and level of reflection are drastically different.

Staining the wells for α-SMA revealed an interesting finding that CTGF could still cause the KO cells to make typical-looking myofibroblasts which were co-incident with the clusters of light reflection (Fig 6). The highly-reflective PDGF-BB cells, however, had a different cellular morphology and while present, the apparent level of α-SMA was drastically lower. This is evidence of a potentially different class of light reflecting cell which may contribute to the overall corneal light reflection.

## Discussion

The model mice tested herein were still able to form sub-epithelial haze with one or both CTGF alleles knocked out. This suggests that CTGF is not necessary for the formation of corneal sub-epithelial haze subsequent to an injury. Fibroblasts from the model mice are highly responsive to PDGF-BB *in vitro*, and the *in vitro* response greatly mimicked the phenomenon observed *in vivo*. PDGF-BB has been found in the tears of patients with corneas healing after photorefractive keratectomy (PRK) surgery[19, 20]. However, the previous reports did not find that tear-borne PDGF-BB levels correlated with fibrosis outcomes. The lack of a relationship between the levels in the tears does not rule out PDGF-BB’s potential role, since no relationship has been established between tear concentrations of a factor and intracorneal concentrations of that factor. Since the cornea is a highly impermeable barrier, no such relationship is reasonably expected. Our data did not resolve the source of residual haze in the mouse corneas, but did demonstrate *in vitro*, a plausible hypothesis centered on the action of PDGF-BB.

For the first time, we have shown here that the cell culture system was capable of demonstrating cellular reflectivity, which is arguably the most clinically-relevant physiological end point in the study of corneal haze formation. To date, past research using *in vitro* work to study growth factors’ roles in the formation of corneal haze have focused on molecular end points including ECM synthesis, actin synthesis and polymerization, and cellular proliferation and migration. On the basis of these molecules, many factors have been found to elicit similar responses. For instance, others have shown that TGF-β_1_ with ascorbic acid can cause cells *in vitro* to recapitulate the collagen III synthesis, actin polymerization, and new lamellae formation[28, 29]. What wasn’t shown in these and other reports are whether these constructs reflect light, and one of the authors have confirmed that they have not looked at it (personal communication with Dr. Karamichos).

In our study, we have shown two commonly studied growth factors were both capable of eliciting a light scattering cellular phenotype *in vitro* which also included α-SMA expression and organization; albeit with a drastically different appearance for both characteristics. At a minimum, these data both support the hypothesis of growth factor redundancy, and give rise to another hypothesis of multiple divergent light scattering phenotypes present in corneal haze. Our findings indicate that CTGF-derived haze and PDGF-BB-derived haze may both be present in normal corneas, and represent 2 of possibly many light reflecting cell phenotypes. Hypothetically, the knockout mice may have solely had non-CTGF derived haze, but may have light scattering cellular phenotypes derived from the actions of other growth factors. Under this hypothesis, one might predict a decrease in haze in the knockout mice since the CTGF-derived haze would be obviated. However, in both the corneas and cells, the light scatter trended towards being greater, evidencing a lack of independence between the two hypothetical modes. Our studies were designed and powered to test solely the hypothesis of CTGF’s necessity for haze, and were therefore underpowered to demonstrate mild modulations in haze level. However, the trend in the data tend to support a the new hypothesis of a potential competition between/among different light scattering phenotypes. Future work using both our new *in vitro* model with growth factors in competition with one another and work with mice with double knockouts would be necessary to test this emergent hypothesis.

Another key finding reported herein is the possible divergence of haze intensity and the quantity of of α-SMA. A recent publication reported efficacy in reduction of α-SMA levels, but no concurrent reduction in haze score by slit lamp[17]. While this observation may be due to the short-comings of using immunofluorescently stained sections (including errors in sectioning plane and the fact that sections represent such a small volumetric sample of the entire wound), the data we have shared here support a possible split between the molecular marker levels and the levels of pathological light scatter. Our data suggest that the cells with the marker do scatter light, but the amount of light scatter is not proportional to the marker itself. Our data do support the continued use of α-SMA as an histological “sign post” for where the light reflecting tissue is. Furthermore, we believe its use is supported for micrographs imaged *en face* as a means to classify cell types based on intracellular organization of α-SMA, as we have done here. Future work may make use of these two applications, when paired with light scatter, to qualify the type of light scattering cells present in a hazy cornea.

## Conclusions

The work reported herein, in combination with our previous work, has demonstrated that CTGF does have a role in corneal wound healing, but that it is more epithelial-centric and not necessary for corneal haze formation. While our data do not support anti-CTGF therapy to prevent haze formation, the results from our other work may indicate that CTGF may be a good target to prevent corneal neovascularization[25]. However, the current most pressing question for any growth factor-centric approach to modulate wound healing is whether there are redundant stimulatory pathways[30, 31], or if each growth factor is responsible for a separate light reflecting cell type. Finally, our work has revealed that while α-SMA has its uses, its use as a surrogate for light scatter is not supported. Once light is shed on this question, more potent and targeted therapies would be more readily designed to account for redundancy, or timed and targeted to the growth factor-specific activities when and where they are needed.

## Supporting information

S1 Dataset CTGF-KO Datasets. (ZIP)
