# Supplementary figures and images for: Connective tissue growth factor is not necessary for haze formation in excimer laser wounded mouse corneas
Source: PLoS One. 2017 Feb 16;12(2):e0172304. doi: 10.1371/journal.pone.0172304 (PMC5313228; doi:10.1371/journal.pone.0172304)

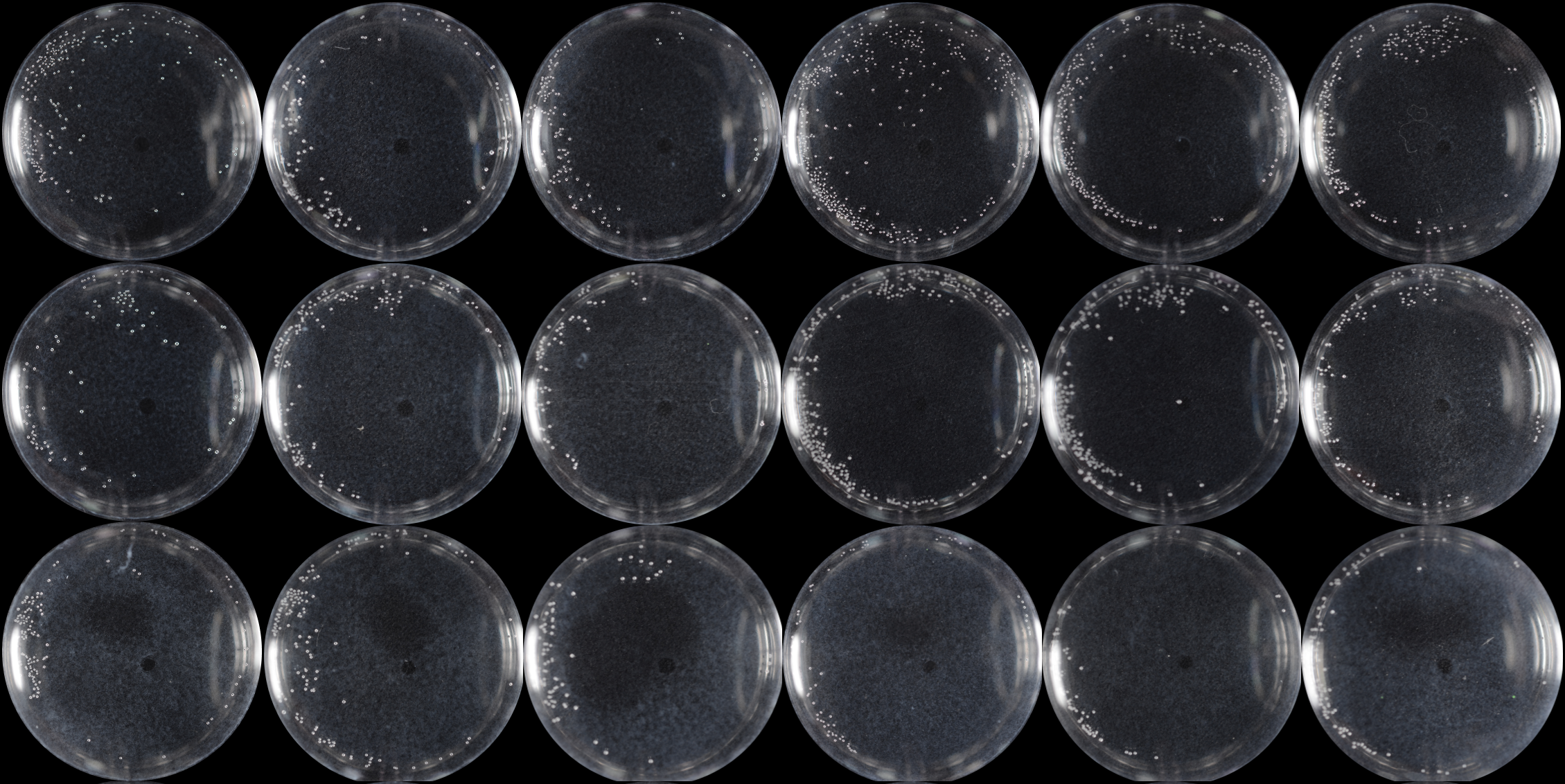

Supplement: S1 Dataset — (ZIP) [file pone.0172304.s001.zip › 24-Well Plate Macro - Plate 3 CTGF KO-MeOH.png]

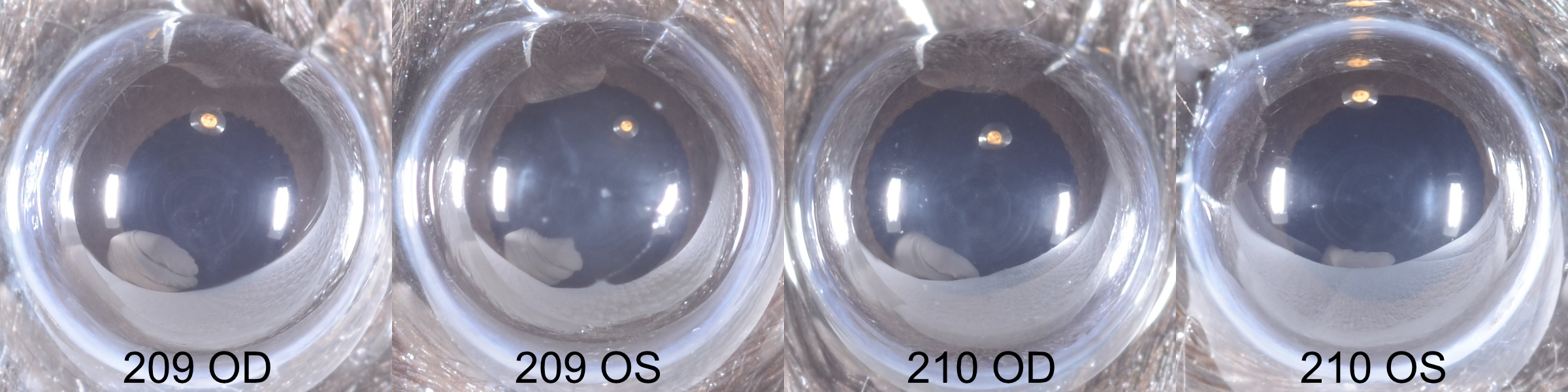

Supplement: S1 Dataset — (ZIP) [file pone.0172304.s001.zip › Full Recombination.png]

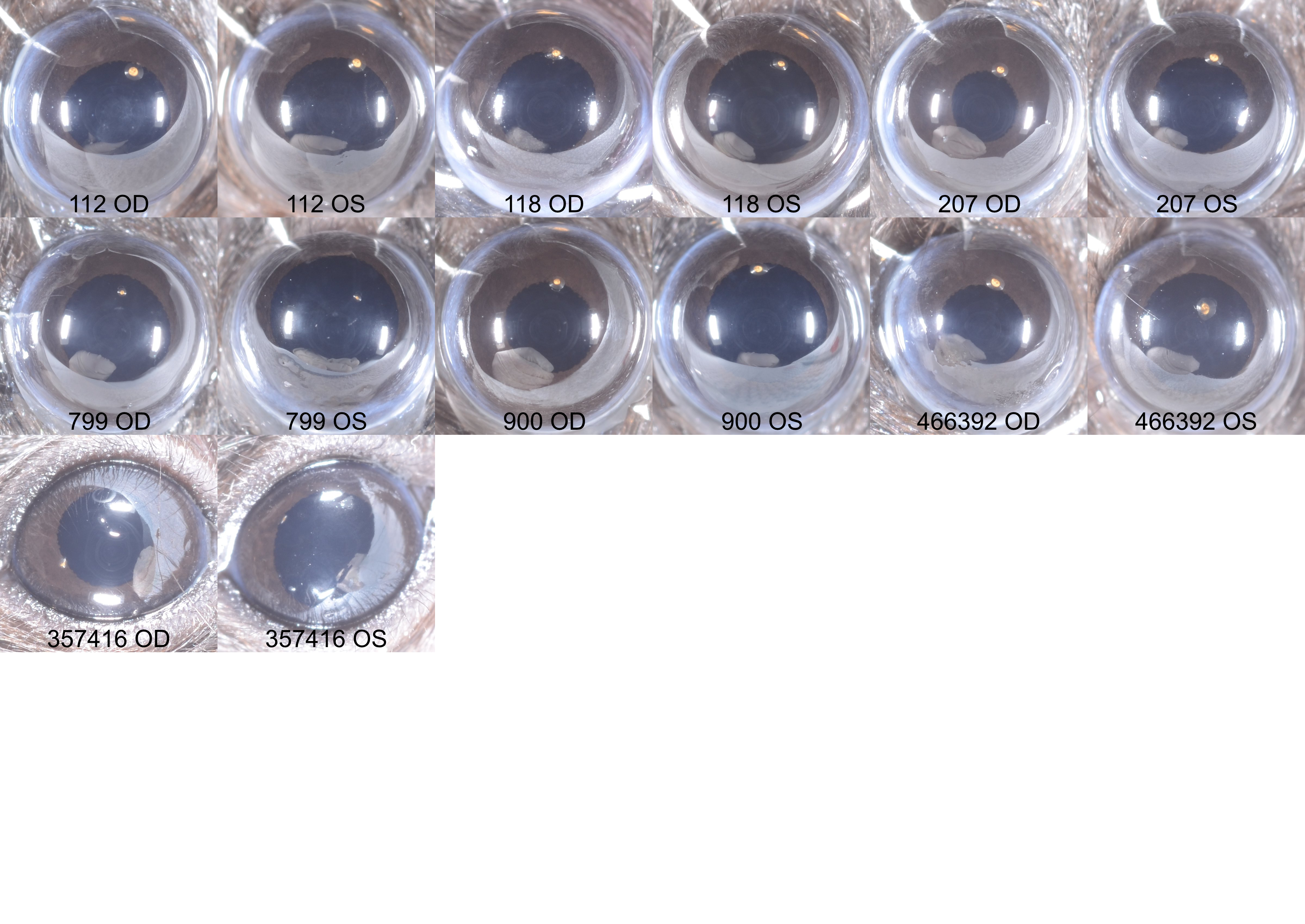

Supplement: S1 Dataset — (ZIP) [file pone.0172304.s001.zip › Grouped-NoRecombination.png]

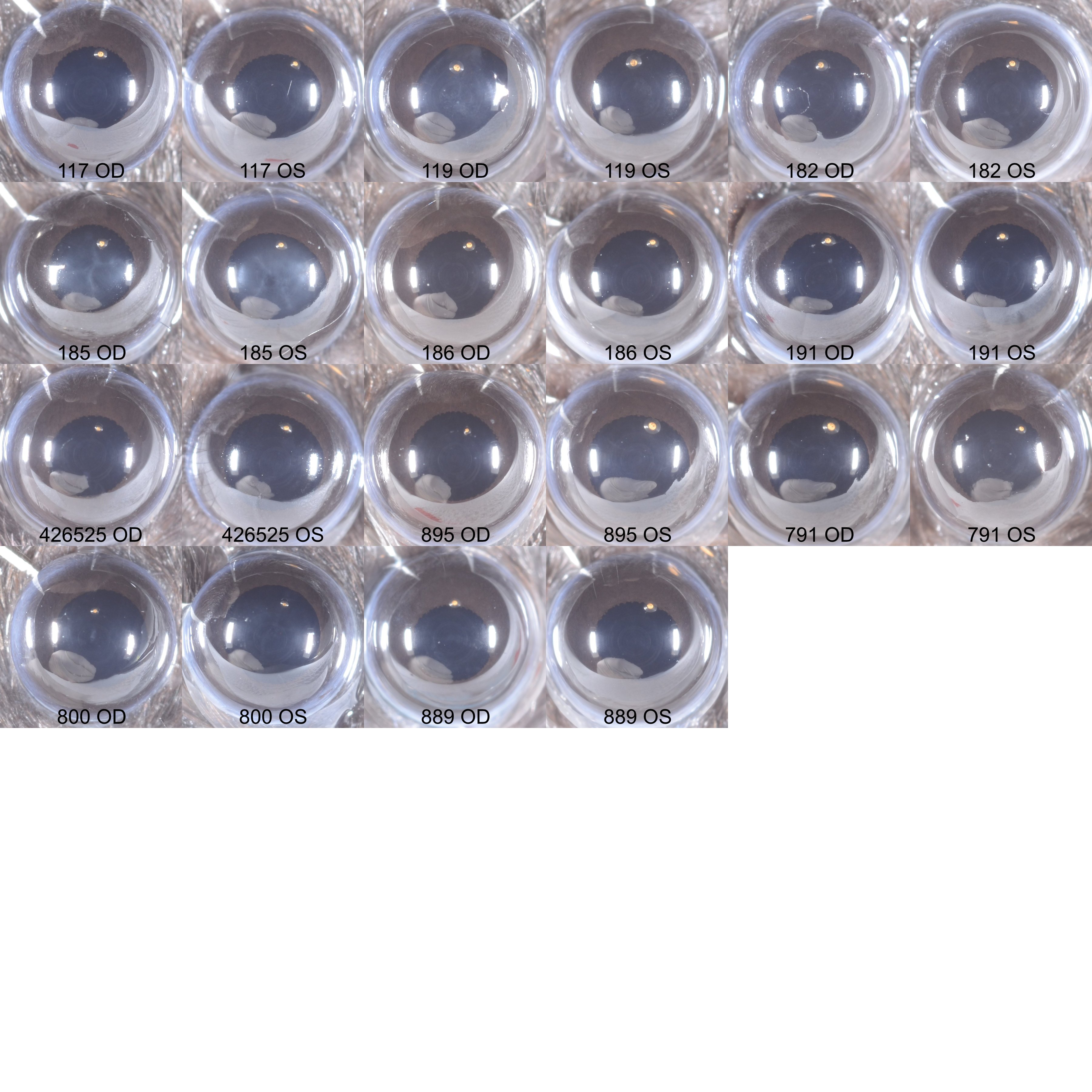

Supplement: S1 Dataset — (ZIP) [file pone.0172304.s001.zip › GroupedHetRecombined.png]
